# Post-traumatic Growth and Related Influencing Factors in Discharged COVID-19 Patients: A Cross-Sectional Study

**DOI:** 10.3389/fpsyg.2021.658307

**Published:** 2021-05-26

**Authors:** Shixin Yan, Jun Yang, Man Ye, Shihao Chen, Chaoying Xie, Jin Huang, Haiyang Liu

**Affiliations:** ^1^Department of Cardiac Surgery, Zhongshan Hospital, Fudan University, Shanghai, China; ^2^Department of Psychiatry, The Second Xiangya Hospital, Central South University, Changsha, China; ^3^Clinical Nursing Teaching and Research Section, The Second Xiangya Hospital, Central South University, Changsha, China; ^4^Xiangya Nursing School of Central South University, Changsha, China; ^5^Changsha Public Health Treatment Center, Changsha, China; ^6^Department of Nephrology, The Second Xiangya Hospital, Central South University, Changsha, China

**Keywords:** post-traumatic growth, COVID-19, post-traumatic stress disorder, social support, self-esteem, coping style

## Abstract

The purpose of this study is to investigate the current state of post-traumatic growth (PTG) and identify its influencing factors in discharged COVID-19 patients. PTG refers to individual experiences of significant positive change arising from the struggle with a major life crisis. This descriptive cross-sectional study used the convenient sampling method to recruit 140 discharged COVID-19 patients in Hunan, China. The results show that the PTG of the discharged COVID-19 patients was positively correlated with self-esteem, post-traumatic stress disorder, coping style tendency, and social support, but negatively correlated with the time from onset to diagnosis. Our findings could provide guidance on improving the psychological state and well-being of discharged COVID-19 patients.

## Introduction

In December 2019, a 2019 novel coronavirus disease (COVID-19) was first reported in Wuhan, China. The disease rapidly became a global pandemic (Li, Q. et al., 2020). The main clinical feature of COVID-19 was diffuse alveolar damage causing acute respiratory failure (Huang et al., 2020). As of March 9, 2021, over 116 million cumulating cases and two million deaths worldwide have been reported to the WHO (World Health Organization, 2021). Inevitably, the rapid spread of COVID-19 resulted in a variety of mental symptoms. In addition to the newly diagnosed COVID-19 patients and those undergoing treatment, the discharged COVID-19 survivors showed psychiatric symptoms, including post-traumatic stress disorder (PTSD), depression, anxiety, insomnia, and obsessive-compulsive symptoms at follow-up (Mazza et al., 2020). However, the psychological factors associated with the post-traumatic growth (PTG) of the discharged COVID-19 survivors have scarcely been investigated. As one of the most discussed positive post-traumatic consequences, PTG refers to an individual's experience of significant positive change arising from the struggle with a major life crisis, and emphasizes the transformation after trauma (Calhoun et al., 2000). All the COVID-19 patients, whether their symptoms are mild or severe, need to be treated and quarantined in hospital. They are not allowed visits from family members. After discharge, they are strictly required to be home quarantined for at least 14 days. This greatly restricts their personal freedom and disrupts all previous lifestyle habits. Hence, COVID-19 is a stressful traumatic event for all patients and survivors. If we can determine the positive psychological outcomes of COVID-19 and their related influencing factors, survivors suffering from psychiatric symptoms may benefit from this finding and get out of the haze.

PTG enables individuals to reframe their experiences and perceive potential benefits from life trauma, resulting in improving their relationships with others, creating new possibilities, advancing personal strength, bringing spiritual change, or increasing the appreciation of life (Jin et al., 2014). Previous studies have found that PTG may occur in various people who have experienced trauma, such as bereavement (Tan and Andriessen, 2021), HIV infection (Ye et al., 2018), combat (Marotta-Walters et al., 2015), earthquake (Ma et al., 2019), and other life-changing events. Studies on patient care indicated that the level of PTG was negatively associated with depressive effect (Siegel et al., 2005), emotional distress (Urcuyo et al., 2005), and positively associated with quality of life (Xiong et al., 2019).

PTSD is a common trauma-related mental disorder, with manifestations that include re-experiencing, avoidance, negative thoughts or moods associated with the traumatic event and hyper-arousal (American Psychiatric Association, 2013). A meta-analysis indicated that PTSD and PTG might co-exist in traumatized people, and the relationship between PTSD symptoms and PTG was more likely to be a curvilinear relationship (Shakespeare-Finch and Lurie-Beck, 2014). This curvilinear relationship can be explained insofar as those reporting PTSD symptoms at intermediate levels reported the highest level of PTG (Butler et al., 2005). Previous studies examining PTSD and PTG focused on people who had experienced natural disasters or chronic illnesses. Therefore, the association between PTSD and PTG in a sample of discharged COVID-19 patients needs further investigation.

Coping refers to the cognitive and behavioral changes brought about by the management of an individual's specific external/internal stressors (Wu et al., 2020). Coping also refers to a style or feature that remains relatively stable under a variety of challenging circumstances (Oldershaw et al., 2009). Coping styles can be divided into two categories: one is a positive response to the active action of stressors (positive coping style), and the other is an adjustment of the emotional state caused by a negative response to stress events (negative coping style) (Compas et al., 1993). According to Tedeschi and Calhoun, coping capacity plays a crucial role in the development of PTG, and some early success in coping was thought to be a precursor to later PTG (Tedeschi and Calhoun, 2004). Studies showed that a higher level of positive coping styles was related to increased levels of positive cognitive and behavioral adjustments in the face of stressful events, thereby reducing the chances of anxiety and of depressive symptoms (Zong et al., 2010; Xiong et al., 2019). Therefore, this study hypothesized that a positive coping style would be associated with the PTG levels of discharged COVID-19 patients.

Social support is also an important influencing factor in PTG (Tedeschi and Calhoun, 2004). Social support can be defined as the extent to which individuals perceive that others around them are available to them and are attentive to their needs (Zysberg and Zisberg, 2020). Social support may increase individuals' self-esteem level, alleviate persistent unpleasant or stressful emotions, and make life more comfortable and meaningful (Lee and Way, 2019), protecting individuals from psychological distress after traumatic events and promoting positive changes after trauma (An et al., 2017; Feng et al., 2018; Karaca et al., 2019). A study conducted during the COVID-19 pandemic suggested that social support could mediate the association between emotional intelligence and worry and that it could play a role in alleviating worry about COVID-19 (Zysberg and Zisberg, 2020). Our study investigated the effect of social support on PTG in individuals who had been hospitalized with COVID-19.

The purpose of this study is to explore the current status of PTG in discharged COVID-19 patients and to analyze its influencing factors. Understanding the potential influencing factors could enable people to determine the better direction needed for psychological counseling after a public health disaster. We hypothesized that mood states, PTSD, coping styles, and social support were correlated with the PTG of discharged COVID-19 patients.

## Methods

### Setting and Participant

This cross-sectional study recruited 140 discharged COVID-19 patients in Hunan Province in February 2020. The inclusion criteria were as follows: (1) Diagnosed according to the COVID-19 Diagnosis and Treatment Regimen in China (5th version); (2) Over 18 years of age; (3) Have normal reading and writing ability, understand the questionnaire content; (4) Can use WeChat related functions correctly; (5) Informed consent of voluntary participation in the study. The exclusion criteria were as follows: (1) Having severe mental disorders; (2) Having organic brain lesions and malignant tumors. The 140 discharged COVID-19 patients included 75 female individuals and 65 male individuals, and the mean age of the participants was (43.47 ± 11.75) years.

### Procedure

A mobile app called “So jump” (www.sojump.com) was used to collect data. This data collection method was chosen to avoid the potential risk of virus transmission during the completion and collection of a paper-based questionnaire. The participants used a mobile phone to scan the QR code on the website and they completed the survey form online. Two trained research nurses supervised the completion of the questionnaires. The 140 questionnaires were distributed and the effective recovery rate was 100%.

### Ethical Considerations

This study was approved by the Medical Ethics Committee of the Second Xiangya Hospital of Central South University (Approval Number: 2020015), in line with the principles embodied in the World Medical Association Declaration of Helsinki. Before the survey began, researchers explained the purpose and significance of the study to the participants. All the participants provided informed consent.

### Measurements

#### General Information Questionnaire

The general information questionnaire was designed by the researchers. It included sociodemographic data (age, gender, education, and place of residence), clinical data (time since discharge, time from onset to diagnosis, clinical classification, comorbidity, and type of infection), and general conditions of participants (self-care ability, activity endurance, sleep quality, hospital-induced panic, and negative effects of COVID-19 on life). In our study, the comorbidities included obesity, hypertension, diabetes, cancer, cardiovascular disease, chronic lung disease, chronic kidney disease, and others. The types of infections were categorized as family clusters or other cases. Family clusters refer to clusters of cases shared in time and location by common exposures within a family. Other cases refer to the sporadic cases, other types of clusters, and community transmission. The self-care ability, activity endurance, and sleep quality were assessed with a 5-point Likert scale, from 1 (“very poor”) to 5 (“very good”). Hospital-induced panic and the negative effects of COVID-19 on life were also assessed with a 5-point Likert scale, from 1 (“none”) to 5 (“very significant”). Hospital-induced panic referred to the feeling of panic at being hospitalized because of COVID-19.

#### Post-traumatic Growth Inventory (PTGI)

The Post-traumatic Growth Inventory (PTGI) was developed by Tedeschi and Calhoun (Tedeschi and Calhoun, 1996) and the Chinese version has shown good reliability and validity in Chinese populations (Ji et al., 2011). There are 21 items divided into five dimensions: relating to others, new possibilities, personal strength, spiritual change, and appreciation of life. Each item is scored on a scale of 0 (“never”) to 5 (“a great degree”). The total score of the PTGI is the sum of all item scores. The normative value of the total score was 49.97 (Ji et al., 2011). A higher score indicates additional positive psychological changes in the aftermath of trauma. Participants were asked to complete the PTGI according to their psychological changes caused by the experience of hospitalization and treatment for COVID-19. In our study, Cronbach's α for this scale was 0.92.

#### Profile of Mood Status (POMS)

The POMS was developed by McNair et al. (1971) and the Chinese version was revised by Zhu (1995). This study adopted the POMS (Chinese version) to assess mood states of discharged patients in the week prior to completing the survey. The scale has 40 items, divided into seven dimensions including tension, anger, fatigue, depression, panic, vigor, and self-esteem. The first five dimensions describe negative emotions, and the other two describe positive emotions. Each item is scored on a scale of 0 (“never”) to 4 (“almost always”). Total Mood Disturbance (TMD) = (Total score of five negative emotions) – (Total score of two positive emotions) +100. The normative value of TMD was 94.45 (Zhu, 1995). A higher TMD score indicates a more negative emotional state. In our study, Cronbach's α for this scale was 0.75.

#### Post-traumatic Stress Disorder Self-Rating Scale (PTSD-SS)

The post-traumatic stress disorder self-rating scale (PTSD-SS, Chinese version) was developed by Liu et al. (1998), who referred to the Post-traumatic Stress Disorder Reaction Index (Pynoos et al., 1993). Each item describes a PTSD symptom, and total of 24 items are divided into five dimensions: subjective assessment of traumatic events (“psychological impact of the disaster”), repeated experience of recurrence (“recurrent dreams related to the disaster”), avoidance symptoms (“avoidance of places or activities related to the disaster”), increased alertness (“sleep disturbance”), and imparirment of social function (“significant impairment of work or study”). Participants were asked to respond based on a 5-point Likert scale ranging from 1 (“not at all”) to 5 (“extremely severe”). The total score for the PTSD-SS is the sum of all the item scores. A high score indicates severe PTSD symptoms. The normative value for the PTSD-SS total score was 34.39 (Liu et al., 1998). In our study, Cronbach's α for this scale was 0.89.

#### Simplified Coping Style Questionnaire (SCSQ)

The Simplified Coping Style Questionnaire (SCSQ, Chinese version) was developed by Xie (1998) based on the Ways of Coping questionnaire by Folkman and Lazarus (1988). Each item describes a coping way, and a total of 20 items can be divided into two dimensions: positive coping (12 items) (e.g., “to be free from work, study, or some other activities”) and negative coping (8 items) (e.g., “relieve trouble by smoking, drinking, taking medicine and holding things”). Participants were asked to agree or disagree on a 4-point Likert scale according to how frequently they adopt each item from 0 (“never”) to 3 (“very often”).

The standard score was used to assess the levels of positive/negative coping manners. The standard score for positive coping style = (the total score for positive coping – the mean value of positive coping)/standard deviation of positive coping style. The standard score for negative coping was calculated in the same way (Dai et al., 2010). The tendency of coping style = the standard score for negative coping – standard score for positive coping (Dai et al., 2010). A tendency score of less than zero, suggests that the subject tends to adopt a positive manner under pressure (Nie et al., 2017). This scale has been commonly used in Chinese, especially during the COVID-19 pandemic (Li, J. et al., 2020; Song et al., 2020; Li et al., 2021; Yao et al., 2021). In our sample, Cronbach's α was 0.90 for the SCSQ, and for two subscales, positive coping and negative coping, it was 0.89 and 0.78, respectively.

#### Multi-Dimensional Scale of Perceived Social Support (MSPSS)

The Multi-Dimensional Scale of Perceived Social Support (MSPSS) was developed by Zimet et al. (1990) and the Chinese version was revised by Kong et al. (2012). Twelve items can be divided into three dimensions: family support, friend support, and other support. Each item is scored on a scale of 1 (“strongly disagree”) to 6 (“strongly agree”). The total score for the MSPSS is the sum of all the item scores. A high score indicates high perceived social support. In our sample, Cronbach's α for the SCSQ was 0.82.

### Statistical Analysis

All analyses were conducted using SPSS 21.0 software (SPSS Inc., Chicago, Illinois). Count data were expressed by frequency and percentage. Measurement data were described by the mean and standard deviation (?x ± s). ANOVA was used to compare the differences in PTG among patients in different groups of categorical variables. Dummy variables were created for all categorical (ordinal) data (Supplementary Table 1). Pearson correlation analysis was used to explore the correlation among variables of general status and psychological status. A stepwise multiple linear regression analysis was used to analyze the influencing factors of PTG. The PTGI total score was modeled as the dependent variable, with general conditions and psychological states as the independent variables. The level of statistical significance was *P* < 0.05.

## Results

### Participant Characteristics

Table 1 shows the sociodemographic and clinical characteristics of discharged COVID-19 patients who participated in the study. The mean age of all participants was 43.47 ± 11.75 years. Most participants were 31 ~ 45 years old. As for the education level, 42.8% of the participants were high school level or below and 57.2% were college degree level or above. The majority of the participants (87.9%) were urban residents. Of the participants, 78.5% had mild and common symptoms, and 76.4% had comorbid diseases, including obesity, hypertension, diabetes, cancer, cardiovascular disease, chronic lung disease, chronic kidney disease, and others. Over half of the participants were cases of the family cluster infections.

**Table 1:** Sociodemographic and clinical characteristics of the participants (*n* = 140).

**Variable**	***N* (%)**	**Post-traumatic growth**	**F/t**	***P***
		**(Mean ± SD)**		
Age (years)^a^			0.58	0.628
≤ 30	23 (16.2)	51.30 ± 19.15		
31 ~ 45	61 (43.0)	50.87 ± 22.58		
46 ~ 60	40 (28.2)	54.80 ± 15.69		
>60	16 (11.3)	47.50 ± 22.02		
Gender			1.58	0.210
Female	75 (53.6)	53.67 ± 20.04		
Male	65 (46.4)	49.38 ± 20.12		
Education level			0.77	0.511
Middle school or below	23 (16.4)	54.26 ± 18.01		
High school	37 (26.4)	54.92 ± 17.31		
College degree	32 (22.9)	49.47 ± 24.89		
Bachelor degree or above	48 (34.3)	49.42 ± 19.67		
Place of residence			5.5	0.020
Urban area	123 (87.9)	50.22 ± 20.37		
Suburban and Rural area	17 (12.1)	62.23 ± 14.70		
Clinical classification^b^			1.84	0.142
Mild	80 (57.1)	50.06 ± 19.55		
Common	30 (21.4)	53.03 ± 18.39		
Severe and Critical	12 (8.6)	62.75 ± 17.43		
Unknown	16 (11.4)	46.50 ± 24.30		
Comorbidity^c^			0.82	0.366
None	107 (76.4)	50.82 ± 20.90		
Yes	33 (23.6)	54.45 ± 17.33		
Type of infection^d^			0.01	0.945
Family cluster	77 (55.0)	51.57 ± 17.66		
Other cases	43 (45.0)	51.81 ± 22.91		

a *The age range of this sample was 21 ~ 69 years old*.

b *According to the COVID-19 Diagnosis and Treatment Regimen in China (5th version)*.

c *Comorbidity included obesity, hypertension, diabetes, cancer, cardiovascular disease, chronic lung disease, chronic kidney disease, and others*.

d *The type of infections were categorized as family clusters or other cases. The family clusters refer to clusters of cases shared in time and location by common exposures within a family. Other cases refer to the sporadic cases, other types of clusters, and community transmission*.

Table 1 also shows the differences in PTG among participants in different groups of categorical variables. Only the patients grouped by place of residence had a significant difference in PTG. The PTG of participants living in non-urban areas was significantly higher than that of participants living in urban areas (62.23 ± 14.7 vs. 50.22 ± 20.37, *F* = 5.50, *P* = 0.02).

### Psychological Status and Correlation Analysis

Table 2 shows the general conditions and psychological status of the participants and the results of the correlation analysis. The time since discharge and time from onset to diagnosis was 21.00 ± 10.00 and 6.41 ± 3.90 days, respectively. The total score for PTG in discharged COVID-19 patients was 51.68 ± 20.12; the scores for the dimensions were as follows: relating to others (7.51 ± 3.43), new possibilities (9.10 ± 4.58), personal strength (8.51 ± 3.58), spiritual change (8.28 ± 3.87), and appreciation of life (18.29 ± 6.78). The total scores of other psychological status factors were as follows: perceived social support (61.90 ± 15.00), TMD (100.44 ± 23.46), PTSD (43.61 ± 17.01), coping style tendency (0.62 ± 1.15). The negative effects of COVID-19 on life, activity endurance, and sleep quality of participants were acceptable (mean score >3).

**Table 2:** Descriptive statistics and correlation.

	**Variable**	**Mean ± SD**	**Range**	**1**	**2**	**3**	**4**	**5**	**6**	**7**	**8**	**9**	**10**	**11**
1	Time since discharge (days)	21.00 ± 10.00	1 ~ 48	–										
2	Time from onset to diagnosis (days)	6.41 ± 3.90	1 ~ 17	0.182^*^	–									
3	Negative effects of COVID-19 on life	3.01 ± 1.16	1 ~ 5	−0.060	−0.066	–								
4	Activity endurance	3.74 ± 0.93	2 ~ 5	0.011	0.057	−0.052	–							
5	Self-care ability	4.93 ± 0.44	1 ~ 5	0.008	−0.013	−0.111	0.094	–						
6	Sleep quality	3.42 ± 1.01	1 ~ 5	−0.009	0.196^*^	−0.131	0.619^**^	0.036	–					
7	Hospital-induced panic	2.43 ± 1.17	1 ~ 5	0.019	−0.126	0.448^**^	−0.087	−0.079	−0.227^**^	–				
8	TMD	100.44 ± 23.46	60 ~ 190	0.006	−0.119	0.376^**^	−0.367^**^	0.025	−0.461^**^	0.578^**^	–			
9	PTSD	43.61 ± 17.01	23 ~ 111	0.053	−0.077	0.348^**^	−0.300^**^	−0.050	−0.504^**^	0.606^**^	0.789^**^	–		
10	Coping style tendency	0.62 ± 1.15	−1.58 ~ 4.76	0.082	−0.121	0.226^*^	−0.210^*^	−0.051	−0.150	0.290^**^	0.443^**^	0.336^**^	–	
11	Perceived social support	61.90 ± 15.00	12 ~ 84	−0.007	0.141	−0.206^*^	0.057	0.012	0.185^*^	−0.174^*^	−0.331^**^	−0.262^**^	−0.338^**^	–
12	Post-traumatic growth	51.68 ± 20.12	0 ~ 97	−0.081	0.015	−0.096	0.009	−0.027	0.074	−0.098	−0.278^**^	0.063	−0.365^**^	0.400^**^

*TMD, Total Mood Disturbance; PTSD, Post-traumatic Stress Disorder*.

* *P < 0.05 (two-tailed);*

** * P < 0.01 (two-tailed)*.

The results of Pearson correlation show that PTG was significantly positively correlated with coping style tendency and perceived social support. PTG was negatively correlated with TMD, indicating that patients with an aversive mood state found it difficult to perceive PTG. In addition, PTSD was positively correlated with hospital-induced panic and mood states, indicating that patients with greater hospital-induced panic and more mood disturbances were more likely to have PTSD symptoms. Multiple variables including activity endurance, sleep quality, coping style tendency, and perceived social support, were negatively related to PTSD.

### Influencing Factors of PTG of Discharged COVID-19 Patients

Table 3 shows a statistically significant regression equation, which explained 42% of the variance in PTG at Block 1. The results showed that TMD, PTSD, coping style tendency, and perceived social support were significantly related to PTG. This suggested that a lower level of mood disturbance, more severe PTSD, more positive coping style, and more perceived social support were associated with a higher level of PTG. Considering that the TMD reflected the overall moods, we further explored the specific mood states that influenced PTG significantly. Therefore, Block 2 was performed. At Block 2, we replaced TMD with the scores for the seven dimensions in POMS. We left other variables unchanged. Two dimensions of POMS, self-esteem and anger were significantly related to PTG. The PTSD, coping style tendency, perceived social support, and time from onset to diagnosis also showed significance at Block 2. This significant regression equation explained 50% of the variance in PTG.

**Table 3:** Multiple regression analyses of post-traumatic growth.

**Variables**	**B**	**SE**	**β**	**t**	***P***
Block 1					
TMD	−0.44	0.10	−0.54	−4.55	< 0.001
PTSD	0.73	0.13	0.64	5.80	< 0.001
Coping style tendency	6.24	1.36	0.36	4.59	< 0.001
Perceived social support	0.29	0.10	0.225	2.30	< 0.001
Adjusted *R*^2^ = 0.42, F = 23.42, *P* < 0.001					
Block 2					
Self-esteem	2.53	0.40	0.48	6.27	< 0.001
Anger	−0.91	0.39	−0.23	−2.32	0.022
PTSD	0.46	0.11	0.41	4.19	< 0.001
Coping style tendency	4.63	1.34	0.27	3.45	0.001
Perceived social support	0.27	0.09	0.21	2.97	0.004
Time from onset to diagnosis	−0.76	0.33	−0.15	−2.29	0.020
Adjusted *R*^2^ = 0.50, F = 21.41, *P* < 0.001					

*TMD, Total Mood Disturbance; PTSD, Post-traumatic Stress Disorder; B, unstandardized coefficients; SE, standard error; β, standardized coefficients*.

## Discussion

To our knowledge, this is the first study exploring psychological PTG and investigating the influencing factors of PTG in discharged COVID-19 patients. Our findings indicated that shortening the diagnosis time, increasing the perceived social support, maintaining a positive coping style, enhancing self-esteem, and easing anger might contribute to PTG.

This study found that the time from onset to diagnosis was negatively correlated with PTG, indicating that shortening this process could help to improve the PTG of patients. The possible reason is that during the time of diagnosis, the patient might be extremely anxious about results, thereby affecting the subsequent treatment and recovery. Early detection by popularizing good information about COVID-19 and early diagnosis by improving the rate of testing could shorten this period of time (Liu et al., 2020).

In this study, perceived social support and positive coping style, as important environmental factors, were found to be positively associated with PTG (Rzeszutek et al., 2017; Peng et al., 2019). According to the model of thriving through relationships presented by Feeney and Collins, social support could provide traumatized individuals with supportive relationships, encouraging them to challenge or extend themselves to grow as individuals, to find goals in life, and to embrace each opportunity to validate their goals, dreams, and aspirations (Feeney and Collins, 2015). The correlation between active mental health and PTG had been reported in a previous study (Sawyer et al., 2010), and the positive coping style that integrated personal mobilization and available resources facilitated active engagement in stressful events and improved positive changes (Stanton et al., 2006). In addition to the positive psychological state of patients, the improvement in the epidemic situation and the development of technology could help to eliminate COVID-19 patients' negative emotions and promote the formation of patients' positive coping styles. Schaefer and Moos proposed a comprehensive model of post-traumatic growth to clarify the factors that contribute to the development of PTG (Schaefer and Moos, 1998). This model implies that environmental resources (e.g., support from family and friends) and personal system factors (e.g., coping style and prior crisis experience) combine to influence event-related factors during a life crisis or a transition period (Schaefer and Moos, 1998). Social support and positive coping styles could influence cognitive appraisal processes and coping responses, influencing, in turn, post-traumatic outcomes (Jia et al., 2015). Based on the results of this study and previous studies, social support and positive coping styles may contribute to the development of PTG.

Among the POMS indicators, self-esteem as the only positive mood state and anger as the only negative mood state, showed a significant correlation with PTG. The positive relationship between self-esteem and PTG was also reported in another study (Lee et al., 2017). Self-esteem is defined as the degree to which people accept and evaluate themselves and obtain a basic sense of self-worth (Dore, 2017). Self-esteem can come from the support of others and their positive evaluation. It can increase confidence in self-ability and self-achievement, and provide discharged COVID-19 patients with more resources to buffer adverse events (Paz et al., 2017; Brunet et al., 2019). Furthermore, self-esteem can provide traumatized people with a high sense of efficacy in coping with difficulties, setbacks, and failures (Mikula et al., 2018), thereby strengthening their use of positive coping strategies to handle negative emotional outcomes (Goodday et al., 2019), leading them to focus more on the positive changes following trauma. However, anger is a distressing affective response commonly observed in persons struggling with traumas. It is necessary to manage distressing emotions and to allow constructive cognitive processing to produce schema changes in the experiencing of PTG (Tedeschi and Calhoun, 2004). In short, enhancing self-esteem and easing anger might be conducive for PTG.

In research on the consequences of traumatic events, the association between PTG and PTSD has been an important issue. Our study found that there was a positive correlation between PTG and PTSD in discharged COVID-19 patients, and those patients with high exposure to PTSD showed higher PTG. Consistent with this result, several longitudinal studies in samples from children and adolescents also reported a positive relationship between the two variables (Wolchik et al., 2008; Kilmer and Gil-Rivas, 2010). Taku et al. in a study of a group of bereaved Japanese university students, also found evidence of a significant positive relationship between PTGI scores and PTSD (Taku et al., 2008). Other similar results have been obtained in US samples (Kilmer et al., 2009) and among children impacted by Hurricane Katrina (Kilmer and Gil-Rivas, 2010). Our study was conducted at an early stage after a traumatic event (21 ± 10 days after discharge). The PTSD symptoms and perceptions of positive post-trauma changes coexisted after a traumatic event, but they were not at opposite ends of a continuum. Individuals actively seek to identify the presence of growth, even amongst ongoing distress, thereby providing the possibility of dealing with even the most severe of challenges, redefining personal strengths, philosophies, and relationships in their future lives (Shakespeare-Finch and Lurie-Beck, 2014). The trend and interaction of PTSD and PTG remain unclear because this study was a cross-sectional study. The development of PTSD and PTG is an ongoing, lifelong process. Future long-term research with follow-ups should observe the relationship between PTSD and PTG and the role of other factors regulating this relationship.

In this study, the results also show that non-urban residents had a higher PTG score than urban residents after discharge from hospital. Similar results had been reported in a previous study (Andrykowski et al., 2017). The reason might be that rural survivors experienced greater distress (Andrykowski et al., 2017) and possessed more “connectedness with nature” linked to greater psychological well-being and meaningfulness than urban survivors (Cervinka et al., 2012). Therefore, if conditions were suitable, urban COVID-19 patients could go to rural areas for recovery after discharge. In addition, the hospital-induced panic was negatively correlated with sleep quality after discharge, affecting coping style tendency and perceived social support. In this public health emergency, the rapidly increasing number of cases in the early stage, the lack of medical resources and the family isolation policies promulgated by the government inevitably caused panic. Psychological panic is an individual's objective response to a major risk event, arising from the personal experience of the perception of risk (Wiegman and Gutteling, 1995). This personal experience could arise indirectly from the media or from other individuals. Therefore, reducing or eliminating hospital-induced panic requires a joint effort by COVID-19 patients and society. In addition, patients should not believe in pessimistic rumors or pay too much attention to negative information. Patients' concerns about disease risk could also be alleviated by positive news and reliable information.

It is noteworthy that we found that discharged COVID-19 patients had similar mental states to those of the general population. In this study, the POMS was adopted to assess the mood states of discharged COVID-19 patients (McNair et al., 1971). The POMS has no specific dimension to assess anxiety. Therefore, we chose the dimensions “tension” and “panic,” closely related to anxiety, to assess the anxiety levels. In POMS, the mean score for tension was 0.79, for panic was 0.73, and for depression was 0.59, suggesting that the overall extent of tension, panic, and depression was between not at all and mild. The percentages of participants with a mean score ≥1 (mild degree) were 37.9% for tension, 35.7% for panic, and 27.1% for depression. Researchers found that about 20 ~ 30% of the general population showed anxiety and depressive symptoms in Italy, Hong Kong, America, the Republic of Ireland, Turkey, and so on (Casagrande et al., 2020; Choi et al., 2020; Forte et al., 2020; Gallagher et al., 2020; Hyland et al., 2020; Özdin and Bayrak Özdin, 2020; Shevlin et al., 2020). These percentages were similar to the percentages in our sample. The negative emotions of discharged COVID-19 patients did not disappear with recovery. First, they had just experienced a dreadful disease, which was highly stressful for mental and physical health. They needed time to process their experiences. Second, after discharge, they were required to be home quarantined for at least 14 days, greatly restricting their personal freedom. They did not get back to normal life quickly. Negative emotions always coexisted with unfamiliar and inadaptable lives. Moreover, they might continue to worry about the likelihood of positive conversion of COVID-19. Therefore, discharged patients still had a degree of anxiety and depression.

The study was subject to the following limitations. First, the relatively small sample size may limit the statistical power, so it is necessary to increase the sample size to validate our results. Second, the proposed model was based on data collected from discharged COVID-19 patients treated in Hunan Province, China. Therefore, the generalization of the results to COVID-19 patients from other areas requires caution. Third, all demographics and major psychological variable assessment data were self-reported by patients, potentially leading to reporting bias. The limitations of the electronic questionnaire meant that we did not ask participants about general conditions with the full classical scales, such as the Athens Insomnia Scale for assessment of the sleep quality (Soldatos et al., 2000) and the Barthel index of ADL for assessment of the self-care ability (Collin et al., 1988). Therefore, these results could only provide a general view of the conditions of the discharged COVID-19 patients. In addition, the study verified the model using cross-sectional data, and the explanation of causal relationships was limited.

PTG was positively related with self-esteem, anger, PTSD, coping style tendency, and social support, but negatively related with anger and time from onset to diagnosis in discharged COVID-19 patients. Those patients with high exposure to PTSD symptoms, strong self-esteem, positive coping styles, higher social support, low level of anger, and short diagnosis time showed a higher level of PTG. Prospective and longitudinal studies of these fields need to be performed to further validate the directionality of our findings and to clarify the influencing factors of PTG.

## Data Availability Statement

The raw data supporting the conclusions of this article will be made available by the authors, without undue reservation.

## Ethics Statement

The studies involving human participants were reviewed and approved by Medical Ethics Committee of the Second Xiangya Hospital of Central South University. The patients/participants provided their written informed consent to participate in this study.

## Author Contributions

SY participated in all aspects of the preparation of this manuscript. JY and SC contributed to the statistical analysis and manuscript revision. MY, SC, and CX performed the sample collection. HL revised the manuscript. HL, JH, and MY contributed to study design and article revision. All authors reviewed the manuscript and approved the final draft.

## Conflict of Interest

The authors declare that the research was conducted in the absence of any commercial or financial relationships that could be construed as a potential conflict of interest.
