# Supplementary material for: Post-traumatic Growth and Related Influencing Factors in Discharged COVID-19 Patients: A Cross-Sectional Study
Source: Front Psychol. 2021 May 26;12:658307. doi: 10.3389/fpsyg.2021.658307 (PMC8189317; doi:10.3389/fpsyg.2021.658307)
Supplement: Supplementary file 1 [file Table_1.DOCX]

**Supplementary Materials**

**Table S1.** The assignments of categorical (ordinal) variables

| Variable | Value |
| --- | --- |
| Gender | Female = 1, male = 2 |
| Age | ≤30 = 1, 31∼45 = 2, 46∼60 = 3, > 60 = 4 |
| Education | Middle school and below = 1, high school = 2, college degree = 3, bachelor degree or above = 4 |
| Self-care ability | Very poor = 1, poor = 2, general = 3, good = 4, very good = 5 |
| Activity endurance | Very poor = 1, poor = 2, general = 3, good = 4, very good = 5 |
| Sleep quality | Very poor = 1, poor = 2, general = 3, good = 4, very good = 5 |
| Hospitalized panic | None =1, mild = 2, moderate = 3, great = 4, very significant = 5 |
| Negative effects of COVID-19 on life | None =1, mild = 2, moderate = 3, great = 4, very significant = 5 |
